# Short term effects of exercise training on exercise capacity and quality of life in patients with pulmonary arterial hypertension: protocol for a randomised controlled trial

**DOI:** 10.1186/1471-2466-11-25

**Published:** 2011-05-23

**Authors:** Louise Ganderton, Sue Jenkins, Kevin Gain, Robin Fowler, Peta Winship, Dianne Lunt, Eli Gabbay

**Affiliations:** 1 School of Physiotherapy and Curtin Health Innovation Research Institute, Curtin University, Perth, Australia; 2 Royal Perth Hospital, Perth, Australia; 3 Lung Institute of Western Australia, Centre for Asthma, Allergy and Respiratory Research, University of Western Australia; 4 Sir Charles Gairdner Hospital, Perth, Australia; 5 School of Medicine and Pharmacology, University of Western Australia, Perth, Australia; 6 School of Medicine, University of Notre Dame, Fremantle, Australia

## Abstract

**Background:**

Advances in the understanding and management of pulmonary arterial hypertension have enabled earlier diagnosis and improved prognosis. However, despite best available therapy, symptoms of exertional dyspnoea and fatigue are commonly reported and result in a reduced capacity to perform daily activities and impaired quality of life. Exercise training has demonstrated efficacy in individuals with other respiratory and cardiovascular diseases. Historically, however, exercise training has not been utilised as a form of therapy in pulmonary arterial hypertension due to the perceived risk of sudden cardiac death and the theoretical possibility that exercise would lead to worsening pulmonary vascular haemodynamics and deterioration in right heart function. Now, with the advances in pharmaceutical management, determining the safety and benefits of exercise training in this population has become more relevant. Only three studies of supervised exercise training in pulmonary arterial hypertension have been published. These studies demonstrated improvements in exercise capacity and quality of life, in the absence of adverse events or clinical deterioration. However, these studies have not utilised an outpatient-based, whole body exercise training program, the most common format for exercise programs within Australia. It is uncertain whether this form of training is beneficial and capable of producing sustained benefits in exercise capacity and quality of life in this population.

**Design/Methods:**

This randomised controlled trial will determine whether a 12 week, outpatient-based, supervised, whole body exercise training program, followed by a home-based exercise program, is safe and improves exercise capacity and quality of life in individuals with pulmonary arterial hypertension. This study aims to recruit 34 subjects who will be randomly allocated to the exercise group (supervised exercise training 3 times a week for 12 weeks, followed by 3 sessions per week of home exercise for 12 weeks) or the control group (usual medical care). Subjects will be assessed at baseline, 12 weeks and 24 weeks.

**Discussion:**

This study will determine whether outpatient-based, whole body exercise training is beneficial and safe in individuals with pulmonary arterial hypertension. Additionally, this study will contribute to clinical practice guidelines for this patient population.

**Trial registration:**

Australia and New Zealand Clinical Trials Register (ANZCTR): ACTRN12609000502235

## Background

Pulmonary arterial hypertension (PAH) is characterised by pathological changes in the pulmonary vasculature that cause an increase in pulmonary vascular resistance (PVR) thereby restricting blood flow through the pulmonary circulation [1,2]. To maintain blood flow, pulmonary artery pressure (PAP) increases [2]. Disease progression leads to right ventricular dysfunction and right heart failure [3,4]. The physiologic response of the pulmonary vasculature to exercise is markedly different from normal in individuals with PAH [1,5]. The pulmonary arteries and arterioles have an impaired capacity to vasodilate and distend, and the increase in cardiac output required to accommodate the metabolic requirements of exercise is limited [1]. As a result, oxygen delivery to the peripheral muscles is impaired [6], contributing to the symptoms of muscle fatigue and dyspnoea [1,5]. Whilst the impairment in cardiac output to meet peripheral oxygen demands during exercise largely contributes to the reduction in exercise capacity [7], skeletal muscle dysfunction may also be implicated in the exercise limitation experienced by individuals with PAH [7-10]. Several studies have demonstrated significant morphological and functional changes in skeletal musculature [8-13], which may be related to the decrease in systemic oxygen transport [7]. Changes such as muscle atrophy, decreased oxidative enzymes and increased proportion of type II muscle fibres lead to the early onset of lactic acidosis and a reduced aerobic capacity [7,9,10]

Despite advances in pharmaceutical therapies, which have resulted in improvements in exercise capacity, haemodynamics and outcome in patients with PAH [14], exertional dyspnoea and fatigue continue to result in difficulty performing activities of daily living, lead to the avoidance of physical activity and adversely impact on health-related quality of life (HRQoL) [15]. This inactivity and the resultant physical deconditioning are likely to further exacerbate the functional limitations associated with PAH [8].

Whilst there is a paucity of studies in the PAH population, the benefits of exercise training in individuals with other cardiopulmonary conditions are well established. Specifically, improvements in symptoms, exercise capacity, peripheral muscle function and HRQoL are well documented following exercise training in chronic left-sided heart failure [16-19] and in chronic obstructive pulmonary disease (COPD) [20-23], populations that both report exertional symptoms similar to PAH. These benefits have been achieved following supervised exercise training, 2 to 3 times per week for a period of 6 to 12 weeks, which comprised endurance exercise at a target intensity of between 40 and 80% of peak exercise capacity (peak oxygen uptake [VO_2_] or maximum heart rate [HR]) [16,17,19-21]. In COPD, programs of 12 weeks duration have been shown to produce greater and more sustained improvements in exercise outcomes when compared to programs of shorter duration [20,21].

Historically, exercise training has been discouraged as a therapy for patients with PAH due to the perceived risk that exercise could lead to sudden cardiac death or worsening pulmonary vascular haemodynamics, acceleration of vascular remodelling and deterioration in right heart function [15]. Some evidence exists to suggest that exercise training can be performed without adverse events or detriment to cardiac function or pulmonary haemodynamics [10,15,24,25], however, the efficacy of exercise training in PAH warrants further investigation [14]. To date, only three prospective studies of exercise training in PAH populations have been published [10,15,25]. These studies recruited subjects with idiopathic PAH or chronic thromboembolic pulmonary hypertension, who were stable on medical therapy and were in WHO functional classes II to IV. The exercise training regimen varied in these studies in terms of the frequency of sessions each week (ranging from 3 to 7), duration (7 to 15 weeks) and the exercise modalities [10,15,25]. The types of exercises performed included cycle ergometry [10,15,25], walking [15,25], peripheral muscle training (i.e. quadriceps) [10], breathing exercises [15,25], upper limb exercises [15,25], yoga [15] and mental conditioning [15]. All three studies demonstrated significant improvements (p < 0.001) in exercise tolerance as measured by an increase in 6-minute walk distance (6MWD) [15,25] or endurance time on a cycle ergometer [10], one study reporting improvements in muscle strength and endurance [10]. Improvements in HRQoL (SF-36 component summary scores) were also reported in one study (p < 0.05) [15]. No adverse events were reported in any of the studies.

The studies to date have demonstrated improvements in exercise capacity in this patient population [10,15,25], however these studies have some limitations in the application of exercise training in Australia. Uchi et al [25] recruited subjects who had recently commenced intravenous PAH therapy and thus it is not possible to differentiate between the benefits of exercise and those attributed to the medication. The training programs have included a variety of modalities such as mental training and yoga [15], interventions that do not form part of most cardiopulmonary rehabilitation programs. Within Australia, most rehabilitation programs designed for cardiac or respiratory populations consist of whole body exercise training and take place in hospital outpatient departments or in community settings [26,27]. This contrasts with the study by Mereles et al [15] in which subjects were admitted for a 3 week inpatient training program prior to performing a 12 week home exercise program. In Australia, the resources for providing inpatient exercise training to individuals who are stable on medical therapy are limited [24]. The study by de Man et al [10] evaluated a 12 week outpatient exercise training program which comprised cycling and quadriceps training. The specificity of lower limb exercise training in this study is likely to account for the large increase in endurance cycle time and may explain the non-significant improvement in 6MWD.

To date, the studies have only reported the immediate effects of exercise training and thus it remains unknown whether the benefits are maintained following cessation of supervised training. It is also unknown whether an outpatient-based, whole body exercise training program is beneficial in patients with PAH and whether this type of program is capable of producing sustained benefits in exercise capacity and HRQoL.

This study aims to investigate the benefits of a 12 week hospital outpatient-based, individualised exercise training program involving supervised exercise sessions, followed by a 12 week home exercise program, for a cohort of subjects with PAH.

We hypothesise that:

(i) A 12 week individualised, whole body exercise training program will improve:

• Exercise capacity (as assessed by peak VO_2_; anaerobic threshold; endurance time measured during the constant-workload cycle ergometry test)

• HRQoL

(ii) Exercise training is safe and improvements in exercise capacity will be achieved without evidence of clinical worsening

(iii) An additional 12 week home exercise program will maintain or further improve the benefits gained during the 12 week, supervised, outpatient program.

## Design/Methods

### Design

This study is a prospective, single blind, randomised controlled trial.

### Participants

Thirty-four subjects will be recruited from the Western Australian State Pulmonary Hypertension Service, at Royal Perth Hospital, Perth, Western Australia.

#### Inclusion/Exclusion Criteria

Subjects will be included in the study if they: have a confirmed diagnosis of idiopathic or familial PAH or PAH associated with connective tissue disorders, based on elevated pulmonary artery pressures measured by right heart catheterisation at rest; are in WHO functional class II or III (receiving PAH-specific pharmaceutical therapy); are medically stable and have been on PAH-specific pharmaceutical therapy for 3 months prior to enrolment into the study, and, are willing to complete the supervised and home exercise training programs. Medical stability will be defined as the absence of recent changes in: specific PAH therapy (no change in sildenafil citrate dose for 6 weeks, no change in endothelial antagonist receptors for 3 months); inotropes (including digoxin) and diuretics; symptoms (including dyspnoea and fatigue); body weight and co-morbid conditions.

Subjects will be excluded if they have: oxygen therapy requirements; significant musculoskeletal disease, claudication pain, neurological or cognitive impairment, psychiatric/psychological or mood disorders that may affect their ability to undertake exercise testing or training; a history of moderate or severe chronic lung disease; cardiac disease associated with cardiac failure, poorly controlled angina, unstable cardiac rhythm; involvement in a supervised exercise training program within the last 12 months.

### Recruitment and Randomisation

**Figure 1:**
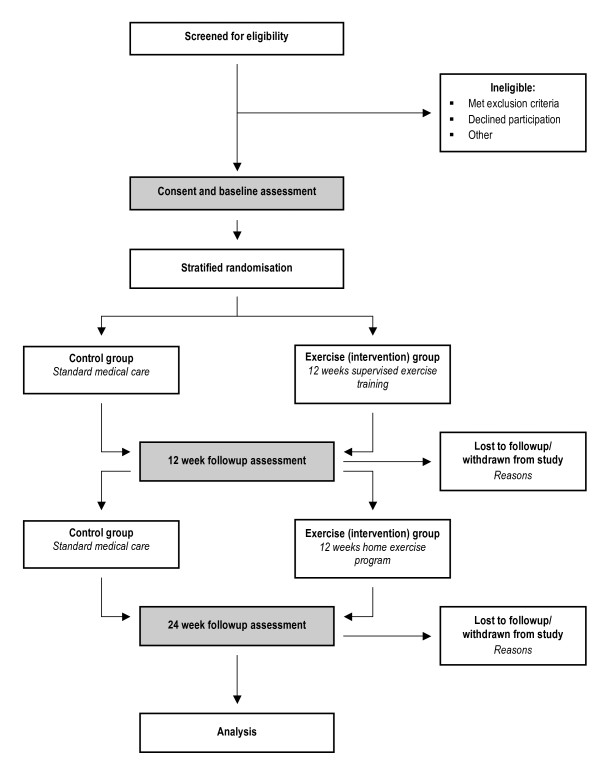
Study design

The flow of subjects through the study is based on recommendations from the Consolidated Standards of Reporting Trials (CONSORT) statement [28] and outlined in Figure 1. Subjects will receive written and verbal information regarding participation in the study and written consent will be obtained from all subjects. The Human Research Ethics Committees of Royal Perth Hospital and Curtin University have approved this study. The study protocol has been registered with the Australian New Zealand Clinical Trials Registry (ACTRN12609000502235).

All subjects will undergo a baseline assessment and re-assessment at 12 and 24 weeks. Following baseline assessment, stratified randomisation to the exercise or control group will be performed. Permuted block randomisation with block sizes of 4 will be used [29]. In order to achieve groups balanced for exercise capacity, subjects will be stratified based on the peak VO_2 _> or ≤ 70% of predicted values [30].

Subjects in both groups will receive usual medical care. Subjects randomised to the exercise group will undergo a 12 week, individually prescribed, supervised, outpatient exercise training program, followed by a 12 week home exercise program.

### Assessment Procedures

Researchers 'blinded' to the subject's group allocation will perform all followup assessments. Each subject will undergo the following procedures at baseline assessment and again at 12 and 24 weeks. Subjects will be required to attend 3 assessment visits within a 2 week period, with each visit separated by at least 24 hours.

#### Visit 1

Subjects will undergo:

(i) Two unencouraged 6-minute walk tests (separated by a rest period of at least 30 minutes) that include monitoring of HR via telemetry (Polar FS3C heart rate monitor, Sportstek, Victoria, Australia), oxygen saturation (SpO_2_) (Respironics 512 pulse oximeter and finger sensor, Philips, Massachusetts, United States) and assessment of symptoms (Borg CR10 [31] dyspnoea and rating of perceived exertion [RPE]).

(ii) Familiarisation with the cycle ergometer and gas analysis equipment.

(iii) HRQoL assessment using the CAMPHOR [32], which has been recently validated in the Australian PAH population [33], and the Medical Outcomes Study Short Form 36 (SF36) Version 2 [34].

(iv) Assessment of physical activity using the International Physical Activity Questionnaire [35].

#### Visit 2

An incremental cardiopulmonary exercise test (CPET) using a continuous ramp protocol (Lode Corival cycle ergometer, Lode, Groningen, Netherlands) [36], to volitional exhaustion with breath by breath gas exchange analysis (Medical Graphics CPX-D, Medgraphics, Minnesota, United States) will be carried out. The test protocol will comprise 3 minutes of upright rest on the bicycle, followed by 3 minutes of unloaded pedalling (0 W). Subsequent workloads will be increased by 5 W/min, 10 W/min or 15 W/min increments depending on the subject's age, gender, weight, 6MWD and diffusing capacity of the lung for carbon monoxide (DLCO) [30]. The aim will be to achieve a test duration of 8 to 12 minutes from commencement of the incremental phase of the test to peak exercise due to volitional task cessation or symptom limitation. Standard criteria will be used to determine if test termination is required for safety reasons [36].

#### Visit 3

A constant workload exercise test will be performed using the same system used for the incremental CPET. The test protocol will comprise 3 minutes of upright rest on the bicycle, followed by 3 minutes of unloaded pedalling (0 W), followed by a step increase in workload to 75% of the peak workload achieved during the incremental CPET. Subjects will be instructed to cycle until voluntary task failure, aiming for a test duration of 6 to 8 minutes. The test will be terminated after 20 minutes of loaded cycling if volitional exhaustion is not reached. Standard criteria will be used to determine if test termination is required for safety reasons [36]. Constant workload cycle tests, at 12 and 24 weeks, will be performed using the identical workload to that used at the baseline assessment.

### Exercise group

Subjects will attend a 12 week, outpatient, individualised, supervised exercise training program involving 3 exercise classes (one hour duration) each week. The sessions will focus on lower limb endurance training (walking and cycling). Lower limb functional strength training (step ups and sit to stands) and endurance training of the upper limbs will also be included with the aim of improving the subject's ability to undertake activities of daily living. Heart rate will be monitored continuously via telemetry (Polar FS3C heart rate monitor, Sportstek, Victoria, Australia) and SpO_2 _(Respironics 512 pulse oximeter and finger sensor, Philips, Massachusetts, United States) prior to and following each exercise. Intensity for the lower limb endurance exercises will be prescribed with the aim of achieving 60-70% HR max (based on age predicted maximum, 220-age [37]), while maintaining SpO_2 _≥ 92% and symptom intensity (Borg CR10 dyspnoea < 4 and RPE < 4). Exercise intensity will be progressed, based on the individual's response to training to maintain HR within the target HR range. The lower limb functional strength training and upper limb endurance exercises will be performed at an intensity that elicits subjective reports of muscle fatigue (RPE 3 to 4) following the completion of 2 sets of 10 repetitions.

#### Home exercise program

The subjects randomised to the exercise group will be asked to commence one session per week of their home exercise program during the last 4 weeks of their supervised program. This is to ensure subjects have the opportunity to discuss the program with the supervising physiotherapist who can assist in troubleshooting any problems that may arise. On completion of the supervised program, subjects will increase the frequency of their home program to 3 sessions per week for an additional 12 weeks. The home program will comprise lower limb endurance training (walking) and lower limb functional strength exercises (step ups and sit to stands). Subjects will be required to record each session on an exercise log. The program will be individually tailored based on the individual's performance during the supervised exercise classes. To guide the home exercise training intensity, symptoms during the supervised period will be correlated to a HR maximum of 120 bpm [15]. Subjects will aim to achieve these symptom responses (Borg CR10 dyspnoea < 4 and RPE < 4) at home to guide exercise intensity.

### Withdrawal Criteria

Subjects will be withdrawn from the study if they demonstrate deterioration in their clinical status (time to clinical worsening), defined by the presence of two or more of the following [38,39]: (i) escalation of medical therapy (increased dose of medication or commencement of intravenous epoprostenol); (ii) deterioration (≥ 1) in WHO functional class; (iii) development of right heart failure (as indicated by increased jugular venous pressure, new/worsening hepatomegaly, ascites or peripheral oedema); (iv) resting echocardiogram changes (deterioration in right ventricular size and function, right atrial size, pulmonary arterial systolic pressure (PASP) and, where PASP cannot be determined, pulmonary acceleration time); (v) oedema that does not respond to oral diuretics; (vi) hospitalisation for PAH; (vii) listing for transplantation; (viii) ≥ 20% deterioration in 6MWD (ix) progressive worsening of dyspnoea at rest or on exertion over the past 3-5 days [40]; (x) deterioration in ventilatory efficiency (V_E_/VCO_2_) reflecting worsening gas exchange.

Subjects may also be withdrawn from the study at their request or if they experience a serious adverse event during or up to 24 hours following an exercise training session and it is deemed unsafe to continue participation by the treating medical officer from the Royal Perth Hospital Pulmonary Hypertension Service. Adverse events comprise: (i) an incident requiring a medical emergency team call (according to Royal Perth Hospital criteria); (ii) exercise-related incident requiring presentation to the Emergency Department or General Practitioner; (iii) hospital admission due to PAH or worsening right heart failure; (iv) an incident resulting in the patient ceasing exercise during class (chest pain, syncope, worsening dyspnoea despite rests, hypotension/hypertension, bradycardia/tachycardia (as measured by a polar monitor and confirmed by palpation), nausea (associated with sweating, pallor and/or tremor), vagueness, vasovagal, arrhythmia); (v) death.

### Outcomes

The primary outcomes of this study will be:

(i) Peak VO_2_;

(ii) Anaerobic threshold;

(iii) Endurance time measured from the constant workload cycle ergometry test;

(iv) HRQoL measured using the CAMPHOR and SF 36 Version 2;

(v) The number of reported acute adverse events (i) reported during exercise training and (ii) resulting in the withdrawal of a subject from the study.

The secondary outcomes of this study will be:

(i) Ventilatory variables at isotime during the constant workload cycle ergometry test (ventilatory equivalent for carbon dioxide [VE/VCO_2_] and end tidal carbon dioxide [PetCO_2_] to describe gas exchange);

(ii) Change in WHO functional class;

(iii) 6MWD (measured from the best of two 6MWTs at each assessment);

(iv) Worsening clinical status (see withdrawal criteria).

### Sample size calculation

Improvements in endurance time following exercise training have recently been reported in the PAH population [10]. These authors [10] reported a mean increase in endurance time, as measured from a constant workload cycle ergometer test, of 270 seconds. The constant workload tests were set at 75% of the peak workload achieved during the incremental CPET, which is consistent with our study's protocol. The mean endurance time (standard deviation) at baseline and post-training were 390 (40) and 660 (60) seconds respectively.

Conservative sample size calculations have been performed using a two-sided Mann-Whitney U test, assuming that the actual distribution is a double exponential. Group sizes of 14 will achieve 91% power to detect a difference of 200 seconds between the null hypothesis that both group means are 600 seconds and the alternative hypothesis that the mean of group 2 is 400 seconds with estimated group standard deviations of 180 and 120 and with a significance level (alpha) of 0.01.

A total of 34 subjects (17 in the exercise group and 17 controls) will be recruited to allow for 20% attrition of subjects from the per protocol analysis.

### Statistical methods

Data will be analysed using SPSS software (Version 17.0; SPSS Inc; Chi). Data that are not normally distributed will be transformed and if transformation fails to satisfy normality, non-parametric tests will be used. The primary and secondary outcomes in the exercise and control groups will be compared using two-way analysis of variance to account for group and time interactions. Statistical relationships between outcome variables will be analysed using Pearson's or Spearman's correlations as appropriate. Data will be analysed using (i) per protocol analysis and (ii) intention-to-treat analysis. Per protocol analysis will be restricted to the data from subjects who complete all assessments and for the exercise group who attend at least 75% of their exercise classes. The required number of sessions must be achieved within a period of 15 weeks for both the supervised and home exercise training programs, allowing for intercurrent illness and other factors that may impact on an individual's ability to attend sessions. The last-observation-carried-forward method will be used to impute missing data at 12 and 24 weeks for the intention-to-treat analysis [41]. The Berger-Exner [42] test will be carried out to determine whether selection bias has been introduced into the study.

## Discussion

This study will be the first randomised controlled trial to evaluate the physiological, clinical and psychological changes that occur following outpatient-based, whole body exercise training in PAH. We hypothesise that exercise training will improve exercise capacity and HRQoL. Adverse events and clinical worsening will be monitored in order to address theoretical safety concerns regarding the potential deleterious effects exercise may have on the pulmonary vasculature and right heart function. Assessment of the subjects following 12 weeks of home exercise program will determine whether the subjects were able to safely maintain the benefits achieved during the supervised exercise training period. Furthermore, this study will contribute to clinical practice guidelines, which will have important implications for the future management of patients with PAH.

## Competing interests

The authors declare that they have no competing interests.

## Authors' contributions

LG, SJ, RF, PW, DL, KG and EG designed this study protocol. LG drafted the manuscript and SJ, RF, PW, DL, KG and EG contributed to the manuscript. All authors have read and approved the final manuscript.

## Pre-publication history

The pre-publication history for this paper can be accessed here:

http://www.biomedcentral.com/1471-2466/11/25/prepub
